# Noise and Financial Stylized Facts: A Stick Balancing Approach

**DOI:** 10.3390/e25040557

**Published:** 2023-03-24

**Authors:** Alessio Emanuele Biondo, Laura Mazzarino, Alessandro Pluchino

**Affiliations:** 1 Department of Economics and Business, University of Catania, 95129 Catania, Italy; 2 Department of Physics and Astronomy, University of Catania, 95123 Catania, Italy; 3 Department of Physics and Astronomy, University of Catania and INFN-Section of Catania, 95123 Catania, Italy

**Keywords:** beneficial role of noise, human stick balancing, financial markets, planetary nervous system

## Abstract

In this work, we address the beneficial role of noise in two different contexts, the human brain and financial markets. In particular, the similitude between the ability of financial markets to maintain in equilibrium asset prices is compared with the ability of the human nervous system to balance a stick on a fingertip. Numerical simulations of the human stick balancing phenomenon show that after the introduction of a small quantity of noise and a proper calibration of the main control parameters, intermittent changes in the angular velocity of the stick are able to reproduce the most basilar stylized facts involving price returns in financial markets. These results could also shed light on the relevance of the idea of the “planetary nervous system”, already introduced elsewhere, in the financial context.

## 1. Introduction

The beneficial role of noise in physics has been explored in several studies, which have shown that a certain degree of noise can enhance the stability or performance of many systems [1,2,3,4,5,6,7]. However, the positive impact of noise is not limited to the physical context but can also be observed in biological systems, such as cognitive processes [8,9,10], neural systems [11,12] or light-harvesting complexes ([13], and in several socio-economic ones, particularly in hierarchical organizations [14], political institutions [15] and financial markets [16,17]. Previous studies in the latter field showed, for example, that random trading strategies can result to be more successful than technical ones [18,19], or that random investments can limit the size of financial bubbles and crashes [20].

The present work further explores this topic by drawing a comparison between two complex systems where noise seems to play a significant role, the human brain and financial markets. This analogy stands on the evidence that both are complex systems at the edge of chaos, i.e., at a critical point in between stability and chaos. Many contributions have consolidated the description of systems lying at the edge of chaos [21], specifically referring to the brain, as pointed out by [22,23,24], and to financial markets [25,26,27,28,29]. In this respect, both these systems are characterized by clear stylized facts, such as the existence of power low distributions [30], which have been identified in neural electrodynamics [31,32] and financial systems [33,34,35].

The leading idea of this study is to envision financial markets as a single system of related markets and comparing it to the Planetary Nervous System: “a goal-oriented, globally distributed, self-organising, techno-social system for answering analytical questions about the status of world-wide society, based on three pillars: social sensing, social mining and the idea of trust networks and privacy-aware social mining” [36]. This technology has mainly been proposed for social network analysis and has never been explicitly used for financial systems analysis. We build upon the hypothesis that financial markets as a whole can be considered as a decentralized planetary nervous system, operating as the human nervous system, where financial market actors act as parts and what happens in one of them causes a reaction in the rest of the system, similarly to what happens in the human body. In addition to the significant role of noise, this analogy has been driven by two other characteristics of financial markets: interconnection and real-time responses.

At present, we live in an increasingly complex and interconnected society, with an enormous amount of data available to us. Observing financial markets, we can find a perfect example of the changes that have taken place in recent decades. In such markets, mainly due to globalization, interconnectedness is extended across territorial borders, market sectors, and through transactional linkages [37,38]. In addition, financial institutions are connected to one another via their counterparty arrangements [39]. Much research has been conducted highlighting this global picture of financial markets. For example, refs. [40,41] examined the interdependence between some international stock indices; Ref. [42] analyzed the effects of South Africa and US shocks on African financial markets; Ref. [43] demonstrated the relationship between the US stock market and the Turkish stock market; Refs. [44,45] showed how US and Japanese stock markets affect Asian countries and [46] found that US equity markets affect world markets and that innovations in the US are rapidly transmitted to other markets. More recently, [47] used networks to measure financial interconnection, such as [48], who used multilayer information spillover networks to measure interconnectedness. Financial markets tend to also be characterized by fairly short reaction times, thus quickly responding to global announcements and information dynamics, such as in the COVID-19 pandemic [49], macroeconomic news [50] or concerning the impact of terrorist attacks on the Istanbul Stock Market [51], sometimes with an immediate market reaction, which, however, recedes almost immediately.

Furthermore, although central coordinating control is missing, financial markets—acting as if they were a global planetary nervous system—try to strive for equilibrium. According to the efficient market hypothesis [52], this tendency is ensured by the fact that arbitrage possibilities are immediately exploited, leading prices back to their fundamental values. Specifically, prices fully reflect all available information and possible variations from the fundamental value can only be traced back to news that are unexpected by agents who, however, respond quickly and rationally by selling and buying until every possibility of profit is canceled out. In this respect, being populated by human beings, each one with their peculiar psychological features, financial markets are also affected by their unpredictable collective behavior, which acts as an endogenous source of noise and contributes to maintain the global system at the edge of chaos. The comparison with the planetary nervous system finds its roots precisely in this connection between the individual human psychology and the financial market trends. Along these lines, the purpose of the present work is that of introducing a tool for studying such a connection. In particular, by means of an “econophysics” approach [53], we challenge the analogy between the idea of a planetary nervous system, operating through the global financial markets, and the individual human nervous system trying to balance a stick on the fingertip.

The idea of balancing unstable objects has long been developed in the field of engineering and control theory. Stabilizing an inverted pendulum is the classical example of control activity over an unstable dynamic system, and several works have been conducted on this; see, e.g., [54,55,56,57,58,59]. A number of authors have conducted studies on the human nervous system in keeping balanced a stick on a fingertip while analyzing statistical properties of finger movements. Pioneering contributions in this field were those by Cabrera and Milton [60,61,62,63,64], who demonstrated, among other things, that fluctuations in the vertical displacement angle of a stick balanced at the fingertip obey a scaling law [60] and that the controlling movements made by the fingertip during the stick balancing can be described by Lévy flight [61]. Refs. [65,66] also focused on the statistical properties of the movements of the stick, while [67] proposed an improvement in stick balancing in the presence of vertical vibration at the fingertip. In a previous enlightening contribution, Ref. [68] underlined some similarities between the time series generated by the experiment of a “balancing stick” presented in [60] and the financial returns.

On the basis of these similarities, we propose a comparison between the distribution of speed changes obtained while trying to balance the stick and the distribution of financial returns. Our results show that, in presence of a certain level of noise, the individual stick balancing phenomenon is able to replicate the main stylized facts of financial markets, namely the appearance of fat tails in the probability density functions of returns [69,70], the absence of autocorrelation [71,72] and the presence of volatility clustering [69]. Our results may be of interest, since they enrich the extant literature on the topic of financial stability with the perspective of human emotional control of investors, which can shed light on implications of financial behaviors beyond orthodox optimization frameworks.

The remainder of the paper is as follows: Section 2 contains the stick balancing model, Section 3 presents numerical results and Section 4 advances some conclusive remarks.

## 2. Materials and Methods

As mentioned in the Introduction, this paper presents a case study of the application of human stick balancing mechanism. In the experiments conducted by [61], six subjects (8–52 years) have been studied while they try to balance sticks with a diameter of 6.35 mm, a mass of 35 g and heterogeneous lengths and material: 39 cm (aluminium) and 62 cm (Garolite). Differences in lengths were analyzed because short sticks are more difficult to balance and require continuous visual feedback control by the nervous system, minimizing the role of proprioceptive inputs.

In their work, they focused on corrective finger movements, interpreting them as a random walk. Authors set the speed, *V*, as the step size per unit time, imaging that the change in speed, ΔV, estimates how fast the hand of the observed subjects can respond to variations in the stick position. This ΔV was characterized in terms of a truncated Lévy flight, with the truncation inversely proportional to skill level increases of the subject involved in the experiment. They measured this level by observing the percentage of times the stick remained balanced for at least 20 s. After two hours of training, three subjects were excluded because they were unable to balance the stick for more than 20 s. Subjects involved in the experiment practiced the stick balancing for 14 h in 10 days and their skills increased during this period, but their nervous system were not capable of predicting the movements of the balanced stick, developing a foraging strategy. Through their experiments, the authors found that most of the times ΔV < 0.5 m/s, although larger changes intermittently occur.

In order to compare the distribution of financial returns with that of speed changes in human stick balancing, we adopted a mathematical model to simulate the stick balancing process. In particular, we follow the works of Cabrera and Milton [60,63], who derived the motion of the stick by numerically integrating the following nonlinear second-order differential equation:

(1) $$\ddot{\theta }\left(t\right)+\frac{\gamma }{m}\dot{\theta }\left(t\right)-\frac{g}{L}sin\theta \left(t\right)+F\left(\theta \left(t\right)\right)=0$$

where *L* is the length of the stick, *g* is the gravitational constant, γ is the damping coefficient, *m* is the mass of the stick and θ is the vertical displacement angle (with θ=0 corresponding to the upright position). The last part of this equation represents the external force that a finger apply on the stick in order to balance it. In real applications, it is necessary to take into account the time required to detect a deviation in θ and, then, to effect a corrective movement. Consequently, the feedback is time-delayed and the ODE in Equation (1) becomes a delay differential equation (DDE):

(2) $$\ddot{\theta }\left(t\right)+\frac{\gamma }{m}\dot{\theta }\left(t\right)-\frac{g}{L}sin\theta \left(t\right)+F\left(\theta \left(t-\tau \right)\right)=0$$

where τ is the time delay, while θ(t) and θ(t−τ) respectively refer to the values of θ at times *t* and t−τ. By expanding *F* as a Taylor series, Equation (2) becomes:

(3) $$\ddot{\theta }\left(t\right)+\frac{\gamma }{m}\dot{\theta }\left(t\right)-\frac{g}{L}sin\theta \left(t\right)+r_{0}\theta \left(t-\tau \right)=0$$

where:

(4) $$r_{0}\left(t\right)=R_{0}+\xi \left(t\right)$$

in which $R_{0}$ is a constant and ξ(t) represents a Gaussian white noise with zero mean and variance σ. This makes the final Equation (3) a stochastic delay differential equation.

A parametric study was preliminarily carried out by numerically integrating [73] Equation (3) in order to find the optimal configuration of parameters able to reproduce the features of human stick balancing.

Some parameters were set and left constant throughout our analysis, since we verified that their influence on the dynamics is limited: they are the damping coefficient γ=0.5 and the initial (small) displacement value θ(0)=0.06. Moreover, we also set g=9.8, m=1 and, at the moment, L=49%, expressed as a percentage with respect to the width of the simulation environment. The parametric study was thus mainly performed by varying $R_{0}$, σ and τ, which are the parameters related to the temporal lag in Equation (3), responsible for the complex dynamical behavior.

A first analysis was carried out in absence of noise, i.e., setting σ=0:

- For τ≤5, there is a critical threshold for $R_{0}$ (specifically $R_{0}^{*}=4.80545$ for τ=0 and $R_{0}^{*}=4.83749$ for τ=5), below which the sticks quickly falls, thus the point identified by the initial conditions θ(0) and $\dot{\theta }\left(0\right)$ in phase space results to be unstable, i.e., a repeller. Above the threshold, the system becomes stable, although the equilibrium point may not coincide with the initial one, and spiral trajectories can be observed approaching the node (in this case, of course, the stick never falls). In other words, in correspondence of the critical threshold $R_{0}^{*}$, there is a sudden transition from a completely disordered regime to a completely ordered one.
- For 5<τ≤10, a third type of regime appears. Below a different critical threshold $R_{0}^{*}$ (which for τ=10 becomes $R_{0}^{*}=4.88424$), we always observe a repeller in the phase space, and the sticks always falls; on the other hand, for $R_{0}^{*}<R_{0}<5$, the stick never falls, and we again find a spiral node; finally, for $R_{0}>5$, the stick falls again, but we now observe a spiral repeller.
- For τ>10 we do not find anymore the regime where the initial point is a spiral node. The stick always falls and we pass from finding a repeller to find a spiral repeller in phase space in correspondence of a critical value starting from $R_{0}∼5$ and increasing as τ increases. For τ=50 and for values of $R_{0}\leq 10$, the transition to spiral repeller is no longer observed.

Summarizing, in the absence of noise, there are different types of transitions from one regime to another, but we always observe either perfectly stable or completely unstable behavior, with dynamical trajectories that appear to be absolutely unrealistic. Actually, in order to reproduce human stick balancing–and, then, financial markets stylized facts—we need an edge-of-chaos-like situation where the stick would obviously fall, but after remaining in equilibrium for a while and showing power–law distributions in velocity changes. In order to obtain such a behavior, we definitely need to add some noise, i.e., we have to set σ>0.

**Figure 1:**
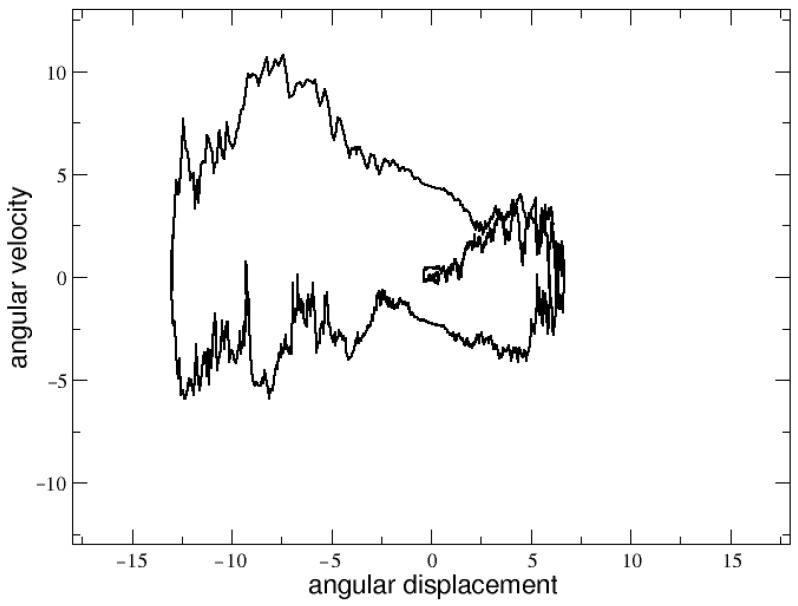
Phase space for τ=2, $R_{0}=5.2$ and σ=10.

Also in the human stick balancing experiments, it has been established that a certain degree of noise is essential for the neural control mechanism [61]. This is a very delicate point, since an overly high level of noise would destabilize the system, and the stick would never balance. Then, after several tests performed by trials and errors (see next section), we arrived at the conclusion that an appropriate level of noise amplitude able to balance the stick corresponds to the value σ=10. Once having fixed this value for σ, we found that whatever the values of $R_{0}$ and τ, the stick falls after intervals of time of different length, which in some cases are enough to allow the appearance of a complex dynamical behavior in phase space. At this level of noise, it is impossible to find exact thresholds between the different dynamical regimes, as in absence of noise. In any case, one can still approximately identify the following behavior:

- For values of τ less than 5, and for $R_{0}$ less than 4, the stick immediately falls for any initial condition, thus showing repeller behavior. Within the small range $4<R_{0}<6$, more interesting dynamics start to be observed in the phase space. An example is shown in Figure 1 for τ=2 and $R_{0}=5.2$. Finally, for larger values of $R_{0}$, the stick still falls but the representative point of the system barely moves from its initial position in the phase space.
- For τ>5, the dynamics start to become very sensitive to the noise, for any $R_{0}$. Generally, as τ increases, a spiral-like repelling behavior emerges for values of $R_{0}>6$. Regardless, for τ∼10 and for $4.5<R_{0}<6.5$, a window of complex behavior does appear, with longer trajectories more suitable for allowing statistical analysis.

**Figure 2:**
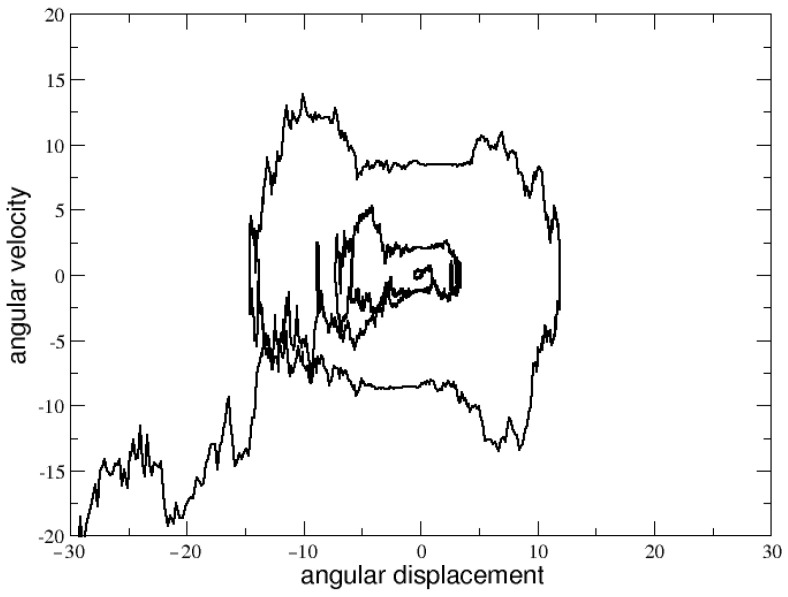
Phase space with τ=10, $R_{0}=5$ and σ=10.

After several trials, we found that, for σ=10 and L=49%, the most interesting and plausible dynamics in the phase space can be found in correspondence of τ=10 and $R_{0}=5$, as shown in Figure 2. In the next section, we will adopt this parameter setting in order to explore the possibility of reproducing financial stylized facts by simulating human stick balancing.

## 3. Results

At first, we had to decide on the number of repetitions of the human stick balancing simulation, obtained by numerically integrating Equation (3), which we should perform for each set of parameters in order to have statistically stable results. Since we did not find significant variations in these results after more than 20 repetitions, for all subsequent analyses, an average of just over 20 runs will be performed. As anticipated in the previous section, we will fix the setting for almost all the main parameters, namely θ(0)=0.06, τ=10 and $R_{0}=5$.

We first checked if the chosen values for the stick length, L=49%, and for the noise amplitude, σ=10, which were determined as suitable in the previous section to achieve a realistic stick balancing dynamics in the phase space, are also suitable for replicating the financial characteristics. In the financial context, the stick length and the noise amplitude could represent a measure of, respectively, the stability of market and random interference of the external socio-economic environment on the feedback mechanism of self-regulation. Specifically, their variation should be restricted within small ranges, in order to better simulate the real features of human stick balancing, where the stick neither immediately falls nor stays balanced for too long time. As we will see in the following, staying within these narrow ranges we will be able to reproduce quite well several stylized facts of financial markets.

**Figure 3:**
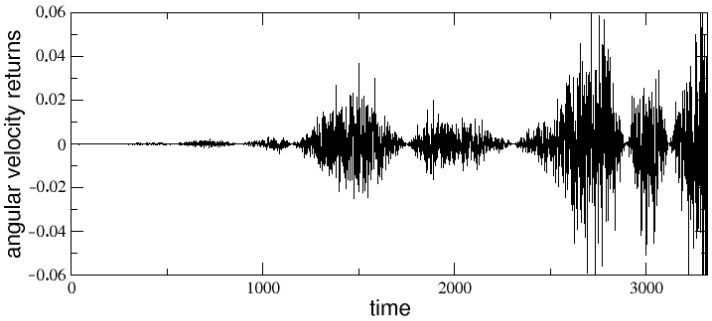
Angular velocity returns as function of time for τ=10, $R_{0}=5$, σ=10 and L=49%.

**Figure 4:**
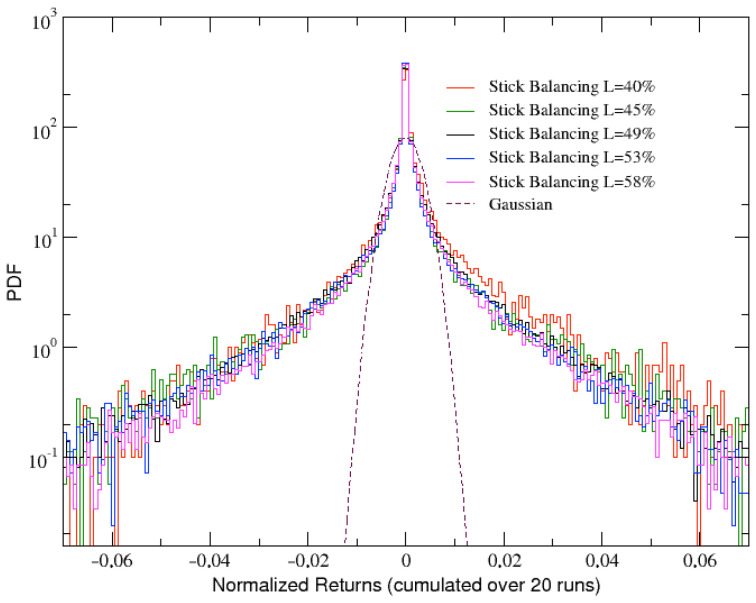
Comparison of several distributions of angular velocity returns (cumulated over 20 runs) at σ=10 and for increasing values of the stick length *L*, expressed in percentage with respect to the width of the simulation environment. Gaussian distribution is also reported as a dashed line for comparison.

Let us start with the analysis of the angular velocity changes, or angular velocity returns, obtained from the stick balancing dynamics and defined as: $\dot{\theta }\left(t\right)-\dot{\theta }\left(t-1\right)$. An example of angular velocity returns time series is shown in Figure 3, which refers to the same run whose dynamics in phase space has been reported in Figure 2. A strongly intermittent behavior generating a fat tailed distribution (PDF) can be immediately noted, as confirmed in Figure 4, where the corresponding PDF is plotted together with the analogous PDFs obtained for other values of the stick length *L*. The curves are also compared with a Gaussian curve (dashed line).

It immediately appears that the tails of the PDFs are slightly wider when the stick is shorter. This is what we actually expect since, when its length is shortened, the stick tends to fall sooner and larger angular velocity changes are needed in order to balance it.

**Figure 5:**
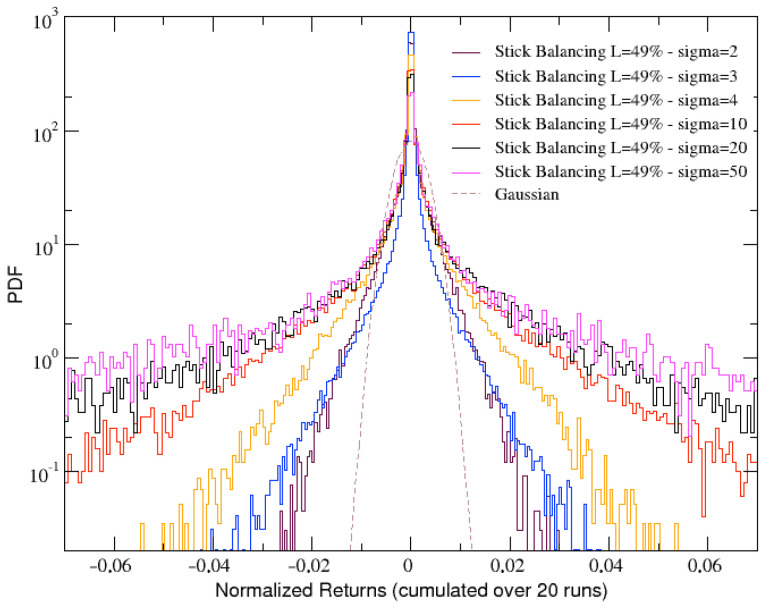
Comparison of several distributions of angular velocity returns (cumulated over 20 runs) at L=49% and for increasing values of noise σ. Gaussian distribution is also reported as a dashed line for comparison.

Focusing on a stick with L=49%, which definitely results as being a good compromise for the stick length, we now compare the angular velocity returns distributions obtained for increasing levels of noise σ. Since increasing σ results in the stick falling more and more quickly, a deeper analysis of the results presented in Figure 5 allow us to conclude that the best compromise between the length of the returns time series (needed for having a good statistics) and the width of the tails of the distributions is obtained for σ=10. Thus, we can also confirm this value as the more suitable for our analysis.

### Empirical Data Collection and Comparison with Simulated Data

We can now proceed with the comparison between the statistical features of our simulated data and those of real financial markets. Financial daily data were gathered for 20 series, including 10 assets and 10 indices. In Table 1 and Table 2 are report, for indices and assets, respectively, the reference periods for each one of the chosen historical series. Data for the assets were collected from Yahoo Finance [74], and data for the indices from the Datastream dataset [75].

**Figure 6:**
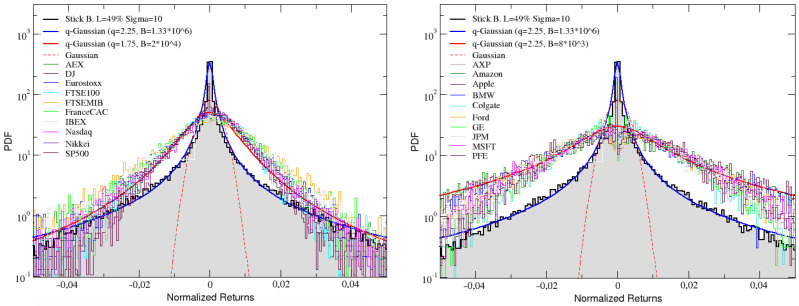
The angular velocity returns distribution of simulated stick balancing, with L=49% and σ=10, is compared with the returns distributions of real financial indexes (**left**) and assets (**right**). In both the panels, Gaussian distribution is also reported as a red dashed line, together with two fitting q-Gaussian curves, reported as blue and red full lines, respectively.

In the two panels of Figure 6, we compare the distribution of returns for the angular velocity obtained simulating a stick balanced on a fingertip with L=49% and σ=10, with the distributions of normalized price returns for the 20 real financial series.

The comparison shows that, indeed, our simulated data replicates the behavior of financial empirical data quite well, since both present similar fat tailed distributions of returns. Such a similarity can be quantified in the context of nonextensive statistical mechanics [76] by fitting both the simulated PDFs and the empirical ones with *q*-Gaussian curves. The latter have the following functional form $y=A{\left(1-\left(1-q\right)Bx^{2}\right)}^{\left(1/(1-q)\right)}$, where the entropic index *q* measures the departure from Gaussian behavior (obtained in the limit q=1 and also reported as a dashed line). When q>1, as typical for financial distributions [77,78,79,80], we are in presence of power–law tails: this is actually what is happening, in our case, for both the simulated and the empirical data (see blue and red full lines, respectively), with values of the entropic index that are exactly the same (q=2.25) for the assets, while being slightly different, but still compatible, for the indexes (also in this case, we could obtain a perfect agreement just by reducing the level of noise—see Figure 5).

**Figure 7:**
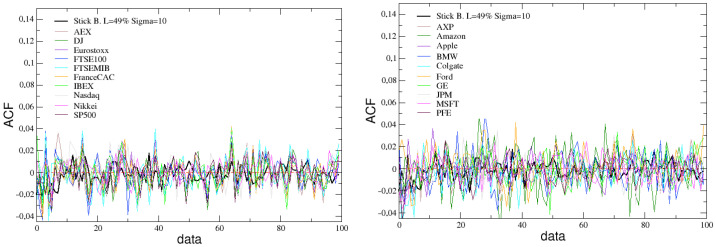
Comparison between the ACF of simulated data, with L=49% and σ=10, and the ACF of real financial indexes (**left**) and assets (**right**).

**Figure 8:**
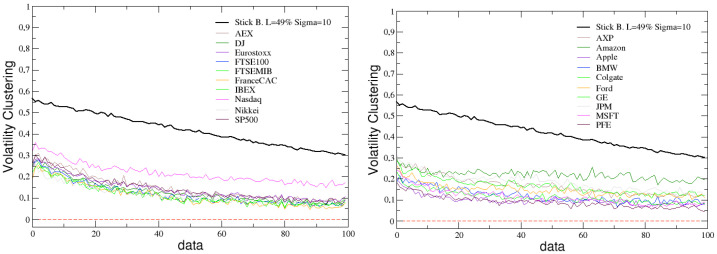
Comparison between the ACF of absolute value of simulated data, with L=49% and σ=10, and the ACF of absolute returns of real financial indexes (**left**) and assets (**right**).

The simulated stick balancing is also able to reproduce other typical stylized facts of financial markets, such as the absence of autocorrelation and the presence of volatility clustering. This can be verified looking at Figure 7 and Figure 8, where these two quantities, calculated for the time series of the angular velocity changes in stick balancing, are reported and compared with the analogous ones calculated for the returns time series of the previously considered indexes and assets.

In Figure 7 the lack of autocorrelation, found in returns of real assets and indices and in our simulated data, implies that it is not possible to forecast price variations, thereby eliminating the potential for simple arbitrage strategies [81]. This reinforces the notion that financial markets are complex systems at the edge-of-chaos, where even small perturbations can result in unpredictable and complex behavior.

Concerning Figure 8, it could be noticed that simulated results seem not able to exactly capture the stationarity of real data, even if a decreasing slope can be appreciated for both indexes and assets. In general, it is not easy to explain the existence of volatility clustering [82], and replicating it by means of a model is difficult and very sensitive to the chosen conditions [83,84,85]. In our case, we are not advancing a specific model of trading (as in [86,87], among others) here. We are more focusing on the identification of a useful predisposition in the statistical properties of a phenomenon linked to the human ability, with the aim of embedding it in a more realistic framework depicting the operation of the market.

## 4. Discussion and Conclusions

This research contributes to a growing body of literature suggesting that noise is not always detrimental, and can in fact play a positive role in several complex systems. In particular, through an extended parametric study performed by numerically integrating a delayed differential equation, we showed that a certain degree of noise is necessary to reproduce a plausible simulation of a human nervous system trying to balance a stick on a finger. Moreover, after proper calibration of the main control parameters, we have also shown that the statistical features of velocity change during the stick balancing process can be successfully compared with the most basilar stylized facts of financial markets, namely the fat tailed distribution of returns, the absence of autocorrelation and the nonvanishing volatility clustering. These results allow us to reinforce the visionary idea of a planetary nervous system (PNS), already proposed by several authors from a different perspective. In analogy with the ability of the individual nervous system in balancing a stick, PNS could actually be invoked here as a possible candidate for representing the hidden mechanism behind the self-regulation ability of global financial markets. In this respect, both human brain and financial markets seem able to benefit from noise for self-maintaining at the edge-of-chaos, a critical state at the border between order and disorder that appears to be essential to ensure their complex performance.

In forthcoming related studies, the potential of the stick balancing model could be further exploited in order to investigate the impact of individual choices on the global stability of financial markets. The role of informative flows on financial equilibria is well known, and as already shown in previous works [87], the excess of information and signals is one of the most relevant channel of transmission of volatility. This is easily confirmed by a vast literature on financial contagion [88,89,90], which can be jointly considered in order to analyze to what extent the individual ability of traders to resist and behave without sharp reactions to any possible small perceived disequilibria can influence the global volatility of markets. While this remains the aim of successive studies, the analogy that we are advancing here between psychomotor skills and psychological factors can represent a first step in the direction of recognizing—and then taming—ingredients of financial instability.

This aspect is important because, as pointed out in the present work, one of the main reasons for market volatility is that financial markets are populated by humans, who are driven by their psychological inclinations. This means that market fluctuations and movements are not solely determined by the dynamics of the true values of the assets but are also influenced by the decisions and the emotions of market participants. It is important to take these human features into consideration when analyzing and predicting market trends. In fact, the value of an asset is determined by a complex web of transactions and signals within the financial market. This can sometimes result in the asset’s value becoming disconnected from its true value. As a consequence, market fluctuations can lead to both profits and losses that do not necessarily reflect the actual value of the company represented by the asset. In this respect, we believe that, despite its present limitations—which we will try to overcome in future analysis—the proposed analogy between financial markets and the stick balancing process could inspire stabilization policies trying to bring the value of the asset back to its “original” value and to avoid the resulting instabilities, such as financial bubbles or crashes.

## Figures and Tables

**Table 1:** Reference periods for real financial indices.

Index	First Day	Last Day
AEX	03/01/1983	06/07/2022
Dow Jones	04/05/1950	06/07/2022
Euro stoxx 50	31/12/1986	06/07/2022
FTSE 100	30/12/1983	06/07/2022
FTSE MIB	31/12/1997	06/07/2022
France CAC 40	09/07/1987	06/07/2022
IBEX 35	05/01/1987	06/07/2022
Nasdaq	05/02/1971	06/07/2022
Nikkei 225	03/04/1950	06/07/2022
S&P 500	31/12/1963	06/07/2022

**Table 2:** Reference periods for real financial assets.

Asset	First Day	Last Day
American Express	12/12/1972	06/07/2022
Amazon	16/05/1997	06/07/2022
Apple	15/12/1980	06/07/2022
BMW	11/11/1996	06/07/2022
Colgate	03/05/1973	06/07/2022
Ford	02/06/1972	06/07/2022
General Electric	03/01/1962	06/07/2022
JP Morgan	18/03/1980	06/07/2022
Microsoft	14/03/1986	06/07/2022
Pfizer	02/06/1972	06/07/2022

## Data Availability

Financial data used for comparison with simulation results are available online at the following links: https://finance.yahoo.com; https://solutions.refinitiv.com/datastream-macroeconomic-analysis.
